# A mixed methods study on evaluating the performance of a multi-strategy national health program to reduce maternal and child health disparities in Haryana, India

**DOI:** 10.1186/s12889-017-4706-9

**Published:** 2017-09-11

**Authors:** Madhu Gupta, Hans Bosma, Federica Angeli, Manmeet Kaur, Venkatesan Chakrapani, Monica Rana, Onno C.P. van Schayck

**Affiliations:** 1 Department of Community Medicine, School of Public Health, Post Graduate Institute of Medical Education and Research, Room No. 130, PGIMER, Chandigarh, India; 2 Department of Social Medicine, CAPHRI, Maastricht University, Maastricht, The Netherlands; 3 Department of Health Services Research, CAPHRI, Maastricht University, Maastricht, The Netherlands; 4 School of Public Health, Post Graduate Institute of Medical Education and Research, Chandigarh, India; 5 Department of Family Practice, CAPHRI, Maastricht University, Maastricht, The Netherlands

**Keywords:** Community intervention, Child health, Inequalities, Mixed methods study, National Rural Health Mission, NRHM, Maternal health

## Abstract

**Background:**

A multi pronged community based strategy, known as National Rural Health Mission (NRHM), was implemented from 2005–06 to 2012–13 in India to curtail maternal and child health (MCH) disparities between poor and rich, rural and urban areas, and boys and girls,. This study aimed to determine the degree to which MCH plans of NRHM implemented, and resulted in improving the MCH outcomes and reducing the inequalities.

**Methods:**

An explanatory sequential mixed methods study was conducted, first to assess the degree of implementation of MCH plans by estimating the budget utilization rates of each MCH plan, and the effectiveness of these plans by comparing demographic health surveys data conducted post (2012–13), during (2007–08) and pre- (2002–04) NRHM implementation period, in the quantitative study. Then, perceptions and beliefs of stakeholders regarding extent and effectiveness of NRHM in Haryana were explored in the qualitative study during 2013. A logistic regression analysis was done for quantitative data, and inductive applied thematic analysis for qualitative data. The findings of the quantitative and qualitative parts of study were mixed at the interpretation level.

**Results:**

The MCH plans, like free ambulance service, availability of free drugs and logistics, accredited social health activists were fully implemented according to the budget spent on implementing these activities in Haryana. This was also validated by qualitative study. Availability of free medicines and treatment in the public health facilities had benefitted the poor patients the most. Accredited Social Health Activists scheme was also the most appreciated scheme that had increased the institutional delivery rates. There was acute shortage of human resources in-spite of full utilization of funds allocated for this plan. The results of the qualitative study validated the findings of quantitative study of significant (*p* < 0.05) improvement in MCH indicators and reduction in MCH disparities between higher and lower socioeconomic groups, and rural and urban areas.

**Conclusions:**

MCH plans of NRHM might have succeeded in improving the MCH outcomes and reducing the geographical and socioeconomic MCH inequalities by successfully implementing the schemes like accredited social health activists, free ambulance services, free treatment and medicines in hospitals for the poor and in rural areas.

**Electronic supplementary material:**

The online version of this article (10.1186/s12889-017-4706-9) contains supplementary material, which is available to authorized users.

## Background

Several safe mother and childhood campaigns that are implemented in developing countries could not reduce maternal and child health (MCH) inequalities successfully, because of either lack of thorough analysis of existing data on inequalities or focus on contextual factors to deal with these inequalities [1]. The status of MCH impact indicators in India, have shown that there is still high infant mortality rate (IMR) of 40 deaths per 1000 live births [2], and maternal mortality ratio (MMR) of 167 per 100,000 live births [3]. Additionally, there is marked difference in the status of MCH outcomes topographically, like lower IMR in urban areas (27 per 1000 live births) as compared to rural regions (44 per 1000 live births) [2]. There is a need to review and evaluate how the current MCH programs are put into effect, so as to produce evidence on the effectiveness of these programs in improving the MCH outcomes and reducing MCH inequalities, as these consume majority of the health budget in developing nations like India. Since implementing a national health program at state and district level is a complex phenomenon, hence to report on its effectiveness the assessment needs to be done both from the health system perspective (supply side) as well as societal (demand side) perspective. Most of the health system assessments are quantitative in nature, and conducting qualitative studies more commonly assesses societal perspectives. Kaur (2016), suggested that mono-methods of assessments might not yield desired results as only quantitative methods may miss contextual information and only qualitative methods may miss to quantify the inequalities [4]. Hence, mixed methods study design is a better study design in such situations, which is being considered in this study for assessing the MCH program in India [5].

National Rural Health Mission (NRHM), a multi pronged community based strategy, was implemented to better MCH outcomes and reduce disparities in MCH than in the past in India, from 2005 to 06 to 2012–13. The aim of this mission was to improve the access to and availability of quality health services, mainly for the poor people so as to reduce inequalities between rich and poor people (socioeconomic disparities); for rural populations so as to reduce inequalities between urban and rural areas (geographical disparities); and for the women and children so as to reduce gender based disparities [6]. The plans of NRHM are described in detail in previously published study protocol [7]. In short, these comprised of health system strengthening by improving the health infrastructure, providing free essential medicines, free patient transport services and medical mobile units to increase the access of MCH services in hard to reach areas; specific MCH schemes included cash benefits to the pregnant women for institutional deliveries, free of cost delivery services in pregnancy and sick neonate’s treatment in public health facilities [8]; and recruiting local village woman known as accredited social health activists, so as to improve the access to domiciliary MCH care in the villages as part of communitization etc. [9, 10]. The goal of NRHM was to reduce the IMR to 30/1000 live births, and MMR to 100/100,000 live births. Most of the earlier evaluations of NRHM were mainly quantitative in nature that lack thorough exploration to identify context specific information regarding varied use of MCH care [11, 12].

The purpose of this study was to ascertain the extent to which MCH plans of NRHM were implemented, and resulted in reducing the MCH disparities and improving the outcomes by mixed methods study design so as to provide explanation for the findings of quantitative study, identify contextual factors for the improvement in MCH outcomes and reduction in MCH inequalities, and enhance or validate the findings of the quantitative study using qualitative study. Such explanations are paramount in understanding the complex implementation process of a national health program at the state and district level, which will guide the policy makers in the effective implementation of the program so as to reach the intended goal of improving the maternal and child survival and reducing inequalities, as second phase of NRHM (2013–2017) is continuing as part of the National Health Mission.

Since the results of the entire mixed methods study was too large to be presented in one paper, and these needed to be presented in detailed manner, hence separate quantitative and qualitative papers, which focused entirely on the quantitative and qualitative study findings, respectively, were published earlier [13–15]. In this paper, findings regarding mixing of the results of quantitative and qualitative study that were not given in earlier papers are presented, after giving the brief reference about quantitative and qualitative studies. Hence, it provides the comprehensive and holistic view of the implementation and effectiveness status of NRHM in Haryana, which only quantitative and qualitative papers earlier failed to provide owing to use of mono-methods.

## Methods

### Study design

**Fig. 1:**
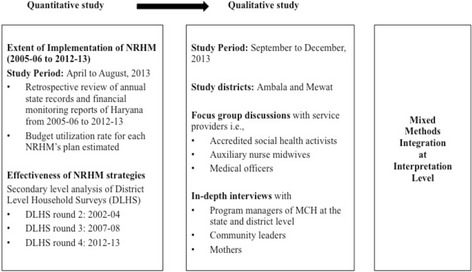
An explanatory sequential mixed methods study design

An explanatory sequential mixed methods study design was used, where in quantitative study was followed by qualitative study, so as to explain the results of quantitative study. [6, 16]. [Fig. 1]. It was a partially mixed design as mixing of the study is done only at the interpretation level.

### Setting

It was conducted in Haryana, a North Indian state. Haryana has 21 districts. Total population in Haryana is 25,353,081 as per census 2011, out of which 70% lives in rural areas. We chose to conduct this study in Haryana as it presents a unique situation, where on one side it is one of the most prosperous state with high per capita income, and on the other side it has several issues like wide differences between and with in districts in terms of having of basic infrastructure, and socio-cultural issues like desire to have preferentially sons, low sex ratio, female feticide, and a low social status of women [17]. Socio-culturally and socioeconomically, it also resembles other North Indian states.

Prior approval of institute’s ethics committee and Government of Haryana was obtained to conduct this study in Haryana.

### Quantitative study

#### Data

Secondary analysis of the data is done for quantitative study by the authors themselves. The annual proportion of sanctioned budget spent under each NRHM’s health plan was used to measure the extent of utilization of budget to implement the health plans, which indirectly provided information on the implementation level of the plans i.e., either fully implemented, partially implemented or not at all implemented. The source of data for sanctioned budget for each MCH plan of NRHM was annual records of proceedings of meetings, which were conducted for approval of state program implementation plan by the central government [18]. The amount of budget spent under each MCH plan of NRHM was obtained from annual financial monitoring reports from 2005 to 06 to 2012–13. Financial monitoring reports were obtained from the state government after prior permission. The methodology of this part of quantitative study is in line with the earlier study on utilization of intergovernmental funds to implement NRHM plans in Haryana [13]. Retrospective review of this data was done from April to August, 2013.

The source of data for assessing the effectiveness of NRHM’s plans in improving the MCH outcomes and reducing the geographical, socioeconomic and gender based MCH inequalities, was nationally representative demographic health survey known as District Level Household Survey (DLHS) for Haryana state. DLHS provide reliable estimates of indicators related to MCH. DLHS round 2 (2002–04) represented the situation before, DLHS round 3 (2007–08) that during and DLHS round 4 (2012–13) that after NRHM implementation. Methodology of these surveys is given in the respective reports of DLHS [19–22]. Sample Registration System’s Bulletin were referred to obtain the information on maternal and infant mortality rates at the Haryana state level [2, 3]. Comparison of status of MCH indicators and disparities, across the socioeconomic, geographical and gender gradients, in the post NRHM’s implementation period was done with the status during and before NRHM implementation by using the DLHS data of three waves (2002–04, 2007–08 and 2012–13). Raw data of DLHS rounds 2,3 and 4 was procured upon request from International Institute for Population Sciences, Mumbai, India [23]. In addition, concurrent evaluation data of NRHM, Haryana (2012–13), was used for obtaining wealth quintile wise information on MCH indicators to estimate socioeconomic inequalities post NRHM, as this was not available in DLHS-4 report [24]. Secondary analysis of DLHS data was done during 2014–15, as DLHS-4 data was not available before this period. The detailed methodology of this part of quantitative study is given in earlier study on effectiveness of NRHM plans in Haryana [14].

#### Data analysis

Statistical Package for Social Sciences (SPSS) version 16 and Microsoft excel was used for data analysis. The proportion of the budget spent out of the allocated budget for implementing a given MCH plan was calculated to estimate the budget utilization rate of each NRHM’s health plan. The degree of implementation of the MCH plans was considered to be fully implemented, if the budget utilization rate was 100% or above, partial if between 1 to 99% and nil if less than 1% at the end of financial year 2012–13. The partial implementation status of the plans was further divided into low- (l9% to 1%), mid- (20% to 79%) and high- (80% to 99%), level. MCH indicators’ trend before-, during- and after- NRHM implementation was observed, and compared using chi^2^ test.

Absolute differences (range) in MCH indicators between poor and rich, rural and urban areas and girls and boys was calculated to assess the socioeconomic, geographical and gender based inequality during three waves of DLHS. It is expected that rate difference score either decreased or reached near to zero after NHRM implementation. The unweighted numbers were available for each respective time period (pre, during and post NRHM) and for the MCH indicators in each group (poor/rich, rural/urban, girls/boys). The reported unweighted numbers and weighted percentages allowed the reconstruction of cross-tabulations (e.g. poor-rich differences regarding institutional deliveries) for the three separate periods. The significant differences (e.g. poor-rich) with in each period were then indicated by Chi^2^ tests.

Logistic regression analysis was done to test the statistical significance of the interaction between the inequality measures (e.g. poor-rich) and period (2012–2013, 2007–2008 and 2002–2004), which indicated whether these differences changed over a time period (e.g. whether the poor-rich difference decreased between 2002 and 2004 and 2012–2013). *P* value was considered significant at the 95% intervals.

### Qualitative study

Qualitative study was done after the completion of NRHM (2005–06 to 2012–13) phase I, i.e., from September to December 2013. To discern which scheme works better in a particular circumstance, extreme case purposive sampling was used to select one well-performing (district Ambala) [25] and one less well-performing district (district Mewat) [26] in terms of status of MCH indicators. Mewat is the least developed district, mostly inhabited by Muslim community, literacy rate of women is quite low, most of the population resides in rural areas and engaged in agriculture, also water is a scarcity here so women spend lot of their time in fetching water and collecting wood for cooking purposes. [27]. Ambala on the other hand is well developed, have high literacy level with better socio-economic conditions [25]. From both these districts we had selected a Community Health Center, a Primary Health Center, a sub-center and village. Forty-one qualitative interviews with 73 participants, including 8 focus group discussions with service providers (medical officers, nurses, accredited social health activists) and 33 in-depth interviews with community representatives, mothers, state and district program managers, were held after obtaining written informed and audio-video consent. Data was collected till saturation, using similar pretested focus group/indepth interview guides [7]. Pretesting of the tool was done in a separate district. Data was collected by two female authors (a research scholar with a Master degree in Public Health and a doctor with MD in Community Medicine), from September to December 2013 (post-NRHM period). These authors were trained in qualitative research methods. In depth interviews and focus group discussions with the participants were conducted in their place of choice. To ensure participants full participation, comfort, privacy and confidentiality, only authors, study staff, and respective participants were present at the time of data collection. The perceptions and beliefs of participants were explored regarding MCH status and disparities between poor and rich, rural and urban areas, or boys and girls and the degree to which MCH plans were implemented. This methodology of the qualitative study is in line with earlier study on qualitative assessment of extent and effectiveness of NRHM in Haryana [15].

#### Data analysis

Data obtained from the qualitative interviews were first transcribed in Hindi language and then translated into English. Codes were identified by two trained independent coders (authors). A conceptual framework of NRHM, as given in Additional file 1: Fig. S1, was used for analysis. According to this framework, NRHM’s health sector plans had four major pillars including health system strengthening, communitization, maternal health care strategies, and child health care strategies; at the base was behavior change communication in the community and women status in general in the society as these were the determinants of MCH outcomes; and together all these would ultimately lead to improvement in MCH and reduction in MCH inequalities. Applied thematic analysis of the content was done to identify the patterns using grounded theory, theory of change and framework approach, in NVivo statistical software version 9. Grounded theory and theory of change was used to construct theories from data and to identify necessary preconditions so as to understand the pathways of change.

### Mixed methods integration

The results of the quantitative and qualitative studies were combined during the interpretation stage in this study, so as to elucidate the results of the quantitative study. Joint display of quantitative and qualitative findings was done for side-by-side comparisons.

## Results

### Quantitative findings

#### Degree of implementation of NRHM health sector plans

The total amount of budget sanctioned for implementing NRHM health sector plans increased from 6.6 million USD in 2005–06 to 66.7 million USD in 2012–13. There was increase in number of community health centers (from 81 to 110), primary health centers (from 408 to 440) and sub centers in the rural areas (from 2433 to 2630) during NRHM implementation in Haryana. Since, scheme wise financial monitoring reports of NRHM’s implementation were available from the year 2007–08 onwards, hence, yearly allocation of funds, expenditure incurred and budget utilization rate under each scheme from financial year 2007–08 to 2012–13 is presented in Additional file 2: Table S1. Based upon the budget utilization rate on various maternal and child health schemes, the extent of implementation of NRHM’s plans is summarized in Additional file 3 **:** Table S2. The trend of implementation status of the four major components of NRHM i.e., health system strengthening, communitization, child and maternal health strategies is presented in Additional file 1: Figure S2. The budget for implementing health system strengthening strategies and communitization was utilized maximally, followed by child and maternal health care strategies. Under health system strengthening component, funds were over-utilized for human resources (110%), patient transport services (115%), and drugs and logistics (170%) in 2012–13. (Additional file 1: Figure S3). The state over-spent on social health activists scheme (133.3%) and patient welfare committees (*Rogi Kalyan Samities*) in the hospitals (112.5%), under communitization component. (Additional file 1: Figure S4). For maternal health care strategies, the budget utilization rate increased steadily for the financial incentive scheme for institutional deliveries (*Jananai Suraksha Yojna*), from 0.8% to 80% from 2007 to 08 to 2012–13. (Additional file 1: Figure S5). The budget utilization rate increased from 66.7% to 106.4% for immunization, and 0% to 37.5% for the integrated management of neonatal and childhood implementation, under child health care strategies. (Additional file 1: Figure S6). For home based newborn care scheme, it drastically increased from 7.7% (2011–12) to 485.7% (2012–13). Additional budget was received from the state health budget.

Based upon the budget utilization rate, it was observed that the patients’ referral transport services, human resources and provision of drugs and logistics under health system strengthening; the accredited social health activists in the villages and patient welfare committee (*Rogi Kalyan Samities*) schemes in the health facilities under communitization; and immunization and home based post natal care under child health care strategies were fully implemented. Most of other schemes were partially implemented.

#### Effectiveness of NRHM health sector plans

**Fig. 2:**
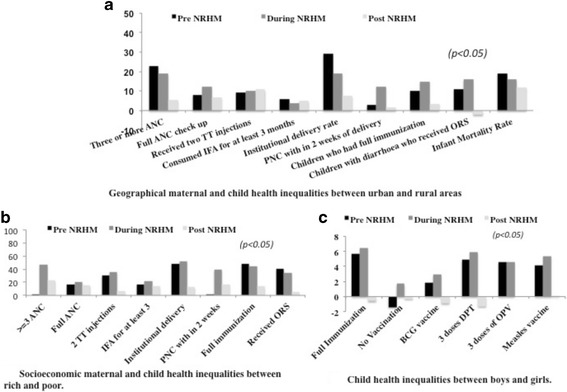
Trend of geographical (**a**), socioeconomic (**b**) and gender (**c**) based inequalities pre during and post NRHM period in Haryana India

Trend of MCH indicators pre, during and post NRHM implementation in Haryana as per DLHS rounds 2, 3 and 4 is given in Additional file 4: Table S3. Significant improvement was observed for most of the MCH indicators after implementation of NRHM. (*p* < 0.05). The IMR had declined from 59 (2002–04) to 40 (2012–13) infant deaths per thousand live births and MMR from 1.86 (2002–04) to 1.21 (2012–13) maternal deaths per thousand live births in Haryana [2, 3, 28]. Significant reduction in inequalities pertaining to various MCH indicators between poor and rich (socioeconomic), rural and urban (geographical), and girls and boys (gender) across time periods (pre-, during and post- NRHM period) was observed, as shown in Fig. 2 and Additional file 5: Table S4. [*p* < 0.05]. Detailed results of the quantitative study are given in earlier studies [13, 14].

### Qualitative findings

The qualitative findings are described in detail in earlier study [13]. Themes codes as per applied thematic analysis are presented in Additional file 6: Table S5. Briefly, it was observed that almost all the stakeholders believed that there was improvement in overall health infrastructure like availability of clean, well-equipped health centers and free medicines in the villages. The reported barriers for having health workforce in sufficient numbers in the government health facilities, particularly in the low performing district Mewat, were frequent transfers of the staff to other districts, unwillingness of the staff to work in rural areas due to lack of facilities like educational, recreational, road connectivity etc., less financial incentives for the doctors to work in difficult areas and no financial incentives for the paramedical staff. Mewat was perceived to be a punishment posting by the health care providers. Accredited social health activists, free referral transport facilities and treatment facilities in rural areas were perceived to have increased the demand and utilization of MCH services, especially institutional delivery, even by the poor families. Functioning of Village Health Nutrition and Sanitation Committees, and celebration of Village Health Nutrition Day reported to be not implemented well. Non-availability of sufficient health care providers had also affected the implementation of various other NRHM schemes like mobile medical unit, free treatment of pregnant woman and infant in the hospital and monthly village health and nutrition day celebration. Overall it was believed that NRHM had a role in reducing socioeconomic and geographical inequalities, and bettering MCH outcomes, but reduction in gender-based inequalities was associated with increase in educational status and acceptance of small family size.

The pathways for change, as derived from theory of change, which might have led to these improvements is given in Additional file 1: Figure S7**,** and is also described in earlier study [13]. An important precondition of improving the maternal mortality ratio and infant mortality ratio was delivery by skilled birth attendant, which was ensured by institutional deliveries. It was observed that the pathway of change which might have led to the increase in institutional delivery included behavior change communication by accredited social health activists in the villages with mothers, availability of free referral transport services and free hospital services for the pregnant women and sick newborns.

### Mixed methods findings

The degree to which MCH plans of NRHM were executed, and succeeded in improving the MCH outcomes and reducing socioeconomic, geographical and gender-based inequality in MCH, is discussed below using the results of quantitative and qualitative study. Joint display of these findings is given in Table 1.

**Table 1:** Joint display of quantitative and qualitative findings regarding extent of implementation and effectiveness of NRHM’s plans in Haryana

A. NRHM’s plans	Extent of implementation		
	Quantitative findings		Qualitative findings
	Budget utilization Rate (%) (2012–13)	Implementation Status	
Health System Strengthening	113.5 ^a^	Full	
Patient transport service/referral services	115^a^	Full	• Free availability of ambulance service • linked to increase in institutional delivery • Issues with its maintenance • late arrivals to the homes • Inadequate number of vehicles
Infrastructure development and strengthening	33.3	Mid level Partial	• Well equipped clean health centers in rural areas available • No waiting halls for patients
Human resources	110^a^	Full	• Acute shortage of manpower especially specialist, • contractual staff available but quality of contractual staff is an issue, • salary of contractual staff not at par with regular staff, • negative attitude of doctors, • specialists not evenly distributed with in the state
Drugs and logistics	170^a^	Full	• Free availability of medicines in health centers in rural areas • quality is an issue; stock out of situations
Mobile medical units	0	None	• non functional mobile medical units • non availability of doctors • limited awareness in the villages
Communitization	121.5^a^	Full	
Accredited Female Health Activist	133.3^a^	Full	• Community Mobilizer • Well known in the villages, has good rapport with the women, especially decision makers (mother in laws) • Role in immunization of children and pregnant women, institutional delivery • Generating awareness about NRHM schemes & importance of institutional delivery • Calls free ambulance and accompanies the families to the hospital for delivery • Insufficient in number • Educational qualification has a bearing on their recruitment
Village health nutrition and sanitation committees	49	Mid level Partial	• Immunization sessions held on village health and nutrition days • Mother’s meetings also held on these days; known popularly as village health ‘*mela’* • Not held regularly • Members are not involved in planning
Village health and nutrition days	0	None	• Less awareness among mothers and community members • Village head would ask for bribe for utilizing the funds • Funds remain unutilized
Maternal health care strategies	58.33	Mid level Partial	
*Janani Suraksha Yojna*	80	High level Partial	• Linked with increase in institutional delivery • Delay in payments due to administrative reasons like opening of bank accounts, proofs required to get the benefits • Lack of knowledge among mothers about this scheme
*Janani Shishu Suraksha Karyakaram*	50	Mid level Partial	• Linked with increased institutional delivery • Free diet during hospital stay available • Lack of adequate manpower
Child health care strategies	91.47	High level Partial	
Facility based new born care	31.3	Mid level Partial	• New born referred for treatment to government hospitals from private health facilities as government new born facilities are better
Integrated management of childhood illnesses	37.5	Mid level Partial	• Trained staff available • Community lack trust on government facilities for treatment of sick children so do not visit health facilities in villages for treatment • Lack of supervision • Poor implementation due to shifting of focus on implementing home based post natal care
Immunization	106^a^	Full	• Lack of sufficient auxiliary nurse midwives • Cultural barrier are there for immunization of children especially in district Mewat • Fear of injections • Accredited social health activists act as an catalyst in acceptance of immunization
B. Effectiveness of NRHM plans in reducing MCH disparities	*P* value ^b^	Statistical significance	Qualitative Findings
Geographical inequality between urban and rural areas	0.00	Significant decline	• Increase in antenatal registrations in rural areas • More villagers utilizing services than urban people due to NRHM. • Awareness has improved and medicines are available in villages however facilities are still more in cities.
Socioeconomic inequality between rich and poor	0.00	Significant decline	• Availability of free ambulances, medicines, diet during hospital stay for the poor. • Food security in general would reduce this.
Gender inequality between male and female child	0.00	Significant decline	• NRHM has no scheme for targeting gender inequality • Small size of the families and increased educational status has led to the changes in gender inequality • Gender inequality is less seen in Mewat district

^a^Extra budget is received from the state budget

^b^ *P* value for difference in the inequality across time periods

#### Health system strengthening

It was observed from the quantitative study that this component was partially implemented. About 50% of the sanctioned budget was spent annually for infrastructure development (Additional file 2: Table S1). Although there was rise in the number of health facilities in the rural areas during NRHM implementation period in Haryana, as observed in the quantitative study, yet the requirement was much more as per the population based health facility norms, as reported in qualitative study. It was also reported that many centers lacked certain diagnostic facilities (in community health centers) and waiting halls for patients (in sub centers and primary health centers).

It was observed that budget on the drugs and logistics was exhausted fully and this component was fully implemented during NRHM implementation, quantitatively. This finding was validated qualitatively. It was reported that free medicines were available in the public health facilities after the NRHM implementation, which had benefitted the poor patients maximally. Patient transport service that was launched during 2008–09, was fully implemented as per the data on budget spent on implementing this activity in Haryana. This was also evident from the qualitative study. The ambulance dial number was widely disseminated among the villagers in the district Ambala. The major factor that had led to rise in institutional delivery rate in the villages was perceived to be due to the availability of free ambulance services, as per service providers and program managers. However, it was also reported that ambulances were slowly breaking down and there was no maintenance or repair of broken down ambulances.

There was gradual increase in budget expenditure on human resources during NRHM implementation (Additional file 2: Table S1). Overall, this component was fully implemented. Qualitatively, it was reported that the availability of doctors, auxiliary nurse midwives, staff nurses increased during the NRHM period (mainly on contractual basis), but simultaneously that the demand of services had increased manifold leading to acute shortages of human resources. Shortages of doctors had also overburdened the existing staff and resulted in a poor access to health care by patients. Implementation of Mobile Medical Units was partial in the state, which is also evident from the results of qualitative study. The medical mobile unit, intended to cater the MCH needs of the hard-to-reach areas in the Mewat district, was believed to be non-functional, possibly due to the lack of doctors.

#### Communitization

Accredited Social Health Activists scheme was fully implemented quantitatively, and was also the most appreciated scheme by all the participants, qualitatively. According to the interviewees, utilization of the ambulance and health facilities by the mothers had also increased through behavior change communication by village health activists. Village health activists were trained in behavior change communication especially in interpersonal communication. They developed good rapport with the families by engaging with the women/their family members/decision makers in their homes, gradually winning the confidence of the families and becoming their confidante. They used the general MCH related information education communication material provided by the state government while interpersonal communication. The implementation of Village Health Nutrition and Sanitation Committee, and Village Health Nutrition Day schemes was observed to be partial, in the quantitative study. Qualitative study findings provided the reasons for the partial implementation of these schemes, like low priority for implementation of these schemes at the state level, poor monitoring and supervision of these schemes, shortage of auxiliary nurses for celebrating Village Health Nutrition Day monthly, and lack of inter-sectoral coordination for utilization of committee’s funds. These findings also corresponded with the budget sanctioned and utilized for implementing these schemes, as shown in Additional file 2: Table S1. For implementing the scheme Village Health Nutrition and Sanitation Committees, no funds were sanctioned till 2011–12, and budget utilization was quite low after that. For Village Health Nutrition Day scheme no funds were sanctioned after 2009–10, even before this period budget utilization vary from 0%–25%.

#### Specific schemes for maternal and child health

Most of the specific MCH schemes (like *Janani Suraksha Yojna, Janani Sishu Suraksha Yojna*) were observed to be partially implemented, except for home based post-natal care and immunization in the quantitative study. During the qualitative interviews it was observed that although increase in institutional deliveries was attributed to schemes like cashless delivery and treatment in the hospitals, and cash payments to the mothers for deliveries in the health facilities, however the funds remained unutilized under these schemes. This was because of linking of funds disbursement with the opening of bank accounts of pregnant women, which had led to issue in delivering the benefits to women who do not have bank accounts. Also proofs were required to get these benefits. Implementation of free hospital delivery scheme was partial due to lack of adequate manpower. In addition, recommended stay in the hospital for a minimum of 48 h after hospital delivery was not possible by many postnatal women, in-spite of the guidelines, due to insufficient number of hospital beds and overriding household responsibilities of the women. Qualitatively, immunization scheme was not fully implemented especially in district Mewat, due to barriers like inadequate number of auxiliary nurse midwives, cultural barriers, fear of injections.

#### MCH indicators and inequalities

Both quantitative and qualitative study results provided evidence that there had been marked improvement in MCH outcome indicators and reduction in geographical and socioeconomic inequalities. Qualitatively, the reason for reduction of MCH inequalities was reported to be due to more awareness regarding MCH services in rural areas by accredited social health activists, availability of medicines and health facilities and increased utilization of MCH services in rural areas, availability of free ambulances, medicines, diet during hospital stay for the poor families.

While quantitative data had shown that there was reduction in the disparity in the immunization status of boys and girls, the qualitative study had provided insight into this change. Injections given for immunization of children were not perceived to be very safe for children; hence, care-takers (especially in the Mewat district) would let girls have it rather than the boys.

“ *We had twin delivery with one boy and one girl in our area. They (the family) breast fed the boy and not the girl. They gave injection (immunization) to the girl but not to the boy. They thought like that, that girl if died of injection would be ok, but boy would be saved.”(*Reported by one of the midwife during FGD with the auxiliary nurse midwives in Mewat*)*

This mixed methods study has highlighted some of the reasons of intra-state differences in MCH improvements and inequalities, even though there is overall improvement in the MCH indicators and reduction in MCH inequalities in Haryana. These reasons include unequal distribution of health care providers with in the districts, non uniform infrastructure development, less number of accredited social health activists, lower level of literacy of women, lower status of women in society, lack of political will in the less performing districts.

## Discussion

This mixed method study has presented the holistic and in-depth review of the extent and effectiveness of NRHM’s MCH plans i.e., health system strengthening, communitization, maternal and child health care strategies in Haryana, by conducting both quantitative and qualitative study. Both quantitative and qualitative studies reported overall partial implementation of NRHM’s MCH plans in Haryana. Quantitative results of improved MCH outcomes and reduction in geographical and socioeconomic MCH inequality were enhanced and validated by the qualitative study [23]. However, the qualitative explanation for improved immunization status among girls than boys as observed in quantitative study was quite a revelation. It was perceived that immunization was unsafe, and hence care-givers would rather let the girl children get it than the boys (especially in district Mewat). The results of the qualitative study also proffered the explanation for the improved MCH outcomes and reduction in inequalities through the construction of pathways of change, and how these could not work in district Mewat, as all the preconditions were not met [23]. It is earlier reported that the mixed methods study design could be especially illustrative, and it could assimilate multiple theoretical frameworks [29], as was done in this study.

Various circumstantial and contextual factors were identified in this mixed methods study regarding degree of implementation of NRHM’s schemes, especially in district Mewat, like acute shortage of human resources in all the health facilities from primary to secondary care level, cultural barriers in accepting immunization/injection etc. Barriers that were reported to prevent the availability of health care providers in this study pointed towards the fact that policies regarding human resource management needed a revamping in the state. The interested local residents from the respective districts should be promoted to acquire the requisite qualification so as to get the job in the health sector in their respective districts. Alternatively, health care providers should be offered the place of posting of their native area. Inequality in distribution of the human resources at the grass root level in the health care delivery system is also reported by Pallikadavath et al. (2013) in India [30]. Availability of primary schools in the villages and good roads were some of the contextual factors that were reported as a determinant factor for the availability of health care providers in their study. Mukherjee et al. (2010) also reported similar inefficiencies in terms of infrastructure and human resource provisions in the states of Jharkhand, Assam, Chhattisgarh and Orissa [10].

The equity issues might be addressed by the program due to better implementation of the schemes like accredited social health activists, free referral transport, financial incentive schemes for institutional delivery or cashless delivery in the public hospital in rural areas, which especially benefitted the poor. The role of accredited social health activists as a catalyst in improving the institutional delivery rate, immunization rate and utilization of various MCH schemes in rural areas was also observed in Uttar Pradesh [31] and Manipur in India [32]. Such role of indigenous community health workers in improving MCH is also reported in other developing countries like Bangladesh [33], and developed countries like Canada among vulnerable groups [34]. Increase in utilization of antenatal care and delivery by skilled birth attendants following implementation of financial incentive scheme (*Janani Suraksha Yojna*) is also reported by Kingkaew et al. (2016) in Myanmar [35]. However, similar barriers in utilizing funds under financial incentive scheme for institutional delivery like the need for having identity documents by pregnant women are also reported by Chaturvedi et al. (2015) in Madhya Pradesh [36]. Overall utilization of certain services, like institutional deliveries, may be improved by cash incentives programs, which are linked to performance of the health workers, if the utilization of these services is evenly low, as reported by Priedman et al. (2013) [37]. However, if the disparities are severe, then such programs without targets will have small effect on reducing disparities. Low performance of village health nutrition and sanitation committees, as observed in this study, is also reported from Maharashtra in India. [38]. At the ground level, NRHM schemes were sometimes considered poorly visible indicating an information gap between service providers and users. Perhaps lessons can be learnt from Taleb et al. (2015) study in Bangladesh, where the maternal and newborn health improved by a focused and dedicated bridging of the information gap through community-based programs that influenced knowledge levels and practices of women [39]. Qualitative findings had also pointed towards the overall socio-political context of a district, especially in district Mewat, in improving MCH. There was less political will to develop district Mewat, probably because of increased allocation of funds to this district due to its underdeveloped status [40, 27].

During 2009–10, NRHM’s plans were reviewed in Jharkhand, Orissa, Assam, Jammu and Kashmir, Uttar Pradesh, Madhya Pradesh, and Tamil Nadu stated using quantitative and qualitative methods by planning commission. MCH services utilization and availability was reported to be improved in rural areas to some extent in that evaluation, and further strengthening of health facilities was suggested [9]. Several limitations of that evaluation were overcome by this study by providing information on the extent of implementation of MCH plans of NRHM, comparing the results with the MCH status before and after the implementation of the NRHM, specifically estimating the MCH inequalities, and assimilating the qualitative and quantitative data by a mixed-methods research.

Review of earlier studies on MCH disparities in India indicated marked MCH inequalities during antenatal, natal or postnatal period to the disadvantage of the poor [41–48]. However, most of these studies reported the status before NRHM implementation period, as observed in this study as well during that period (2002–04). Since this study compared the MCH status and inequalities over NRHM implementation period, hence it provides information on the status of MCH disparities and outcomes during and after NRHM period in Haryana, North India, as well. It also provides tailor-made solutions to further bridge the MCH related gaps, in Haryana (especially in district Mewat). This is pertinent in the context of considering state as unique entity having its own sociocultural background and issues. However, this does not limit the lessons learnt from this study to Haryana, as these may also be exchangeable to other states in India because of having similar health care delivery systems with in the states.

Using a mixed methods study design is the strength of this study, as quantitative assessment of community’s needs and status as per the providers’ perspective was complimented by assessing the felt needs of the health service users by conducting qualitatively study. Thus a mixed methods approach brought the users’ perspective to the fore [4, 5, 49]. Joint display of quantitative and qualitative data helped us in understanding how mixed methods design provided new insight into the implementation process of NRHM’s MCH plans [50, 51]. Investigation for community health research by using mixed methods research design is not only an effective way of research, but is also a foundation for primary care research [52, 53]. This is probably the first study that has evaluated the national program using mixed methods approach in Indian settings, and has provided useful insight and explanations for the findings of the quantitative study.

Having said so, there is definitely a scope for improvement in future for carrying out such studies. In-depth review of how funds are being spent on implementing the NRHM’s scheme in the district, right to the village level, can give us better understanding of the process of budget expenditure on implementing programs at various health care delivery levels. This information will bring further clarification on barriers and facilitating factors to improve the implementation. We also acknowledge that the budget left unspent may not indicate true implementation status of a given NRHM plan, because the reasons why it could not be spent fully might be due to more efficient use of funds or too high estimation on the forehand, hence this aspect was investigated qualitatively as well so as to have better insight of how spending of the money is appreciated and judged by the key persons involved.

The causal association between NRHM implementation and MCH outcomes and inequalities however, cannot be established, as there was no control region (without NRHM). There might have been other (confounding) developments in the same time period (e.g. improving socioeconomic conditions in general) that brought up the positive changes.

The results of this study has an implication for policy makers on the way the program is implemented during the second phase of NRHM as part of National Health Mission. Appreciable achievements were observed for schemes (patient ambulance services, cashless hospital delivery, cash payments for deliveries in the health facilities, and village health activists) that had intended to raise the rate of deliveries by skilled birth attendants among the poor and rural women during the first phase of NRHM implementation. Hence, it is recommended that these schemes should be further strengthened by effectively tackling the reported bottle necks for the implementation of these schemes like increasing the number of ambulances per district especially in district Mewat, placing a maintenance mechanisms for the ambulances, improving the availability of human resources like doctors, nurses, auxiliary nurse midwives, and social health activists, and preventing administrative delays in providing incentives to the pregnant women; and strengthening the supportive supervision of child health plans including integrated management of neonatal and childhood illness.

Another challenge that needs to be addressed is of the long-term sustainability of financial incentive schemes or free MCH services in the government hospitals under NRHM. If these schemes are no longer remain sustainable or government feels burdened then is there a possibility that MCH status or inequalities worsens. However, there is less likelihood of such situation in India, as Indian economy is on the rise [54], and it has committed to increase health expenditure on health from the current gross domestic product (GDP) of 1.15% to 2.5% GDP by 2025, as per National Health Policy 2017 [55].

In future, a structured mixed methods approach may also be used in the planning phase of a rigorous community-based participatory research program so as to develop acceptable, community need based MCH interventions for vulnerable populations (especially for districts like Mewat) to meet the desired MCH goals [56].

## Conclusions

It can be concluded that NRHM’s plans might have succeeded in improving the MCH outcomes, and in reducing geographical and socioeconomic inequalities in Haryana by successfully implementing accredited social health activists scheme, free referral transport scheme, free medicines and providing maternal and child health schemes for the poor and rural women. However, the decline did not reach the intended goal (which was a decrease to a maternal mortality ratio of less than 1 per 1000 live births and a decrease of infant mortality rate to 30/1000 live births) may be due to partial implementation of NRHM’s schemes. Gender-based inequalities are linked to increased education level and adoption of small family norms. Underlying socio-economic development of the district was observed to be an important determinant of MCH (from district Mewat’s experience). Health policy makers should take this aspect into account while framing future policies related to MCH. Overall it can be said that Haryana is on the right track for achieving the sustainable development goals of reducing MCH inequalities.

## Additional files

Additional file 1: Figure S1. The conceptual Framework of NRHM. **Figure S2.** Comparison of budget utilization rate of health system strengthening, communitization, maternal and child health care strategies components of National Rural Health Mission from 2007 to 08 to 2012–13. **Figure S3.** Trend of budget utilization rate of strategies under health system strengthening component of National Rural Health Mission from 2007 to 08 to 2012–13. **Figure S4.** Trend of budget utilization rate of strategies under communitization component of National Rural Health Mission from 2007 to 08 to 2012–13. **Figure S5.** Trend of budget utilization rate of maternal health care strategies of National Rural Health Mission from 2007 to 08 to 2012–13. **Figure S6.** Trend of budget utilization rate of child health care strategies component of NRHM from 2007 to 08 to 2012–13. **Figure S7.** The pathway of change as derived from theory of change. (PDF 300 kb)

Additional file 2: Table S1. Year wise distribution of budget sanctioned, expenditure incurred (in million USD) and percentage of budget left unspent for NRHM’s maternal and child health sector plans from the financial year 2007–08 to 2012–13. (PDF 113 kb)

Additional file 3: Table S2. Extent of Implementation on NRHM’s maternal and child health sector plans in Haryana. (PDF 69 kb)

Additional file 4: Table S3. Status of maternal and child health indicators pre, during and post NRHM implementation in Haryana as per DLHS rounds 2, 3 and 4. (PDF 106 kb)

Additional file 5: Table S4. Geographical, socioeconomic and gender inequalities in maternal and child health indicators pre, during and post NRHM implementation (expressed as absolute difference in proportion of indicators). (PDF 106 kb)

Additional file 6: Table S5. Themes and codes as per applied thematic analysis. (PDF 81 kb)

## References

[1] Say L, Raine R (2007). A systematic review of inequalities in the use of maternal health care in developing countries: examining the scale of the problem and importance of the context. Bull World Health Organ.

[2] Registrar General of India. Sample Registration System. Available at censusindia.gov.in/vital_statistics/SRS_Bulletins/SRS%20Bulletin%20-Sepetember%202014.pdf. Accessed 7 Sept 2017.

[3] Registrar General of India. Special Bulletin on Maternal Mortality in India. Sample Registration System 2011–13. Available at censusindia.gov.in/vital_statistics/mmr_bulletin_2011-13.pdf. Accessed 10 September 2015. Acecssed 7 September 2017.

[4] Kaur M (2016). Application of mixed method research in public health. Indian J Comm Medicine.

[5] Creswell JW (2015). A concise introduction to mixed methods research.

[6] Hota P, Dobe M (2005). National rural health mission. Indian J Public Health.

[7] Gupta M, Angeli F, van Schayck OC, Bosma H (2015). Effectiveness of a multiple strategy community intervention to reduce maternal and child health inequalities in Haryana, North India: a mixed methods study protocol. Glob Health Action.

[8] *Janani Shishu Suraksha Karayakaram.* National Health Mission. Ministry of Health and Family Welfare. Government of India. Available at http://nhm.gov.in/janani-shishu-suraksha-karyakram.html. Accessed 7 July 2017.

[9] Ministry of Health and Family Welfare. National Rural Health Mission. Framework for implementation. Government of India. New Delhi. 2005. Available at http://www.nipccd-earchive.wcd.nic.in/sites/default/files/PDF/NRHM%20-%20Framework%20for%20Implementation%20-%20%202005-MOHFW.pdf. Accessed 7 Sept 2017.

[10] Ministry of Health and Family Welfare. Government of India. National Health Mission. 2013. Available at http://www.pbnrhm.org/docs/mission_doc.pdf. Accessed 7 Sept 2017.

[11] Planning Commission. Evaluation study of National Rural Health Mission (NRHM) in seven states. Government of India: Programme Evaluation Organisation; 2011. Available at http://planningcommission.nic.in/reports/peoreport/peoevalu/peo_2807.pdf. Accessed 7 Sept 2017.

[12] Mukherjee S (2010). A study on effectiveness of NRHM in terms of reach and social marketing initiatives in rural India. Eur J Sci Res.

[13] Gupta M, Angeli F, Bosma H, Prinja S, Kaur M, van Schayck OC. Utilization of intergovernmental funds to implement maternal and child health plans of a multi-strategy community intervention in Haryana, North India: a retrospective assessment. PharmacoEconomics Open. 2017; 10.1007/s41669-017-0026-3. 10.1007/s41669-017-0026-3 PMC5711747 29441503

[14] Gupta M, Angeli F, Bosma H, Rana M, Prinja S, Kumar R, van Schayck OC (2016). Effectiveness of multiple-strategy community intervention in reducing geographical, socioeconomic and gender inequalities in maternal and child health outcomes in Haryana. India PLoS one.

[15] Gupta M, Bosma H, Angeli F, Kaur M, Chakrapani V, Rana M, van Schayck OCP (2017). Impact of a multi-strategy community intervention to reduce maternal and child health inequalities in India: a qualitative study in Haryana. PLoS One.

[16] Leech NL, Onwuegbuzie AJ (2009). A typology of mixed method research designs. Qual Quant.

[17] Economic Survey of Haryana. Department of Economic and Statistical Analysis Haryana. Government of Haryana. 2012–13. Available at http://www.esaharyana.gov.in/Data/Economic%20Survey%20of%20Haryana/2012-13.pdf. Accessed 7 Sept 2017.

[18] National Rural Health Mission. Record of Proceedings to approve program implementation plans (PIP) of Haryana. 2005–12. Available at http://www.nrhmharyana.gov.in/files/ROP%202008-09.Pdf Accessed on 7 September 2017.

[19] International Institute of Population Sciences. District Level Household and Facility Survey-2. Haryana Report. Reproductive and Child Health Project. Ministry of Health and Family Welfare. New Delhi. 2002–04.

[20] International Institute of Population Sciences. District Level Household and Facility Survey-3. Haryana Report. Reproductive and Child Health Project. Ministry of Health and Family Welfare. New Delhi. Haryana. 2007–08.

[21] International Institute of Population Sciences. District Level Household and Facility Survey-4. State Fact Sheet Haryana. Reproductive and Child Health Project. Ministry of Health and Family Welfare. New Delhi. 2012–13.

[22] International Institute of Population Sciences. District Level Household and Facility Survey-4. Haryana Report. Reproductive and Child Health Project. Ministry of Health and Family Welfare. New Delhi. 2012–13.

[23] International Institute for Population Sciences, Mumbai, India. Online data request. Available at http://www.iipsindia.ac.in/. Accessed 7 Sept 2017.

[24] School of Public Health. Post Graduate Institute of Medical Education and Research (PGIMER). Chandigarh. Concurrent Evaluation of National Rural Health Mission. Haryana. 2013–14.

[25] International Institute of Population Sciences. District Level Household Surveys. Fact Sheet District Ambala. Reproductive and Child Health Project. 2012–13.

[26] International Institute of Population Sciences. District Level Household Surveys. Fact Sheet District Mewat. Reproductive and Child Health Project. Ministry of Health and Family Welfare. New Delhi. India. 2012–13.

[27] Niti Ayog. Government of India. Identifying backwardness of Mewat region in Haryana: A block level analysis. 2015. Available at http://niti.gov.in/writereaddata/files/document_publication/Identifying%20Backwardness%20of%20Mewat%20Region%20in%20Haryana-%20A%20Block%20Level%20Analysis_final_0.pdf. Accessed 7 Sept 2017.

[28] Registrar General of India. Maternal and Child Mortality and Total Fertility Rates. Sample Registration System. 2004–06, 2007–09. Available at http://censusindia.gov.in/Vital_Statistics/SRS_Bulletins/Final-MMR%20Bulletin-2007-09_070711.pdf. Accessed 7 Sept 2017.

[29] Wesely PM (2010). Language learning motivation in early adolescents. J Mix Methods Res.

[30] Pallikadavath S, Singh A, Ogollah R, Dean T, Stones W (2013). Human resource inequalities at the base of India's public healthcare system. Health Place.

[31] Jain N, Srivastava NK, Khan AM, Dhar N, Adish V, Menon S (2008). Assessment of functioning of ASHA under NRHM in Uttar Pradesh. Health Popul Perspect Issues.

[32] Sapril L, Richards E, Kokho P, Theobald S (2015). Community health workers in rural India: analysing the opportunities and challenges accredited social health activists (ASHAs) face in realizing their multiple roles. Hum Resour Health.

[33] Rahman M, Jhohura FT, Mistry SK, Chowdhury TR, Ishaque T (2015). Assessing community based improved maternal neonatal child survival (IMNCS) program in rural Bangladesh. PLoS One.

[34] Smylie J, Krist M, McShane K, Firestone M, Wolfe S, O’Campo P (2016). Understanding the role of indigenous community participation in indigenous prenatal and infant-toddler health promotion programs in Canada: a realist review. Soc Sci Med.

[35] Kingkaew P, Werayingyong P, Aye SS, Tin N, Singh A (2016). An ex-ante economic evaluation of the maternal and child health voucher scheme as a decision-making tool in Myanmar. Health Policy Plan.

[36] Chaturvedi S, De Ayesha C, Raven J. Does the *Janani Suraksha Yojna* cash transfer program to promote facility births in India ensure skilled birth attendance? A qualitative study of intrapartum care in Madhya Pradesh. Global Health Action. 2015;8:27427. doi:10.3402/gha.v8.27427 10.3402/gha.v8.27427 PMC4497976 26160769

[37] Priedeman SM, Curtis SL, Basinga P, Angeles G (2013). An equity analysis of performance-based financing in Rwanda: are services reaching the poorest women?. Health Policy Plan.

[38] Sah PK, Raut AV, Maliya CH, Gupta SS (2013). Performance of village health, nutrition and sanitation committee: a qualitative study from rural Wardha, Maharashtra. The Health Agenda.

[39] Taleb F, Perkins J, Ali NA, Capello C, Muzahid A, Santarelli C, et al. Transforming maternal and newborn health social norms and practices to increase utilization of health services in rural Bangladesh: a qualitative review. BMC Pregnancy and Childbirth. 15(75) doi:10.1186/s12884-015-0501-8. 10.1186/s12884-015-0501-8 PMC4391089 25886165

[40] Institute for Human Development. Ministry of Minority Affairs, Government of India and Indian Council of Social Science Research. A baseline survey of minority concentration districts of India. Available at http://icssr.org/Overview%20Report.pdf. Accessed 7 Sept 2017.

[41] Pathak PK, Singh A (2011). Trends in malnutrition among children in India: growing inequalities across different economic groups. Soc Sci Med.

[42] Prusty RK, Kumar A (2014). Socioeconomic dynamics of gender disparity in childhood immunization in India, 1992-2006. PLoS One.

[43] Pathak PK, Singh A, Subramanian SV (2010). Economic inequalities in maternal healthcare: prenatal care and skilled birth attendance in India, 1992-2006. PLoS One.

[44] Pathak PK, Singh A, Subramanian SV (2015). Economic inequalities in maternal health care: prenatal care and skilled birth attendance in India, 1992-2006. PLoS One.

[45] Singh A, Pallikadavath S, Ram F, Ogollah R (2012). Inequalities in advice provided by public health workers to women during antenatal sessions in rural India. PLoS One.

[46] Singh A, Padmadas SS, Mishra US, Pallikadavath S, Johnson FA, Matthews Z (2012). Socio-economic inequalities in the use of postnatal care in India. PLoS One.

[47] Pradhan J, Arokiasamy P. Socioeconomic inequalities in child survival in India: A decomposition analysis. Health Policy. 2010; doi:10.1016/j.healthpol.2010.05.010. 10.1016/j.healthpol.2010.05.010 20576309

[48] Goli S, Doshi R, Arokiasamy P (2013). Pathways of economic inequalities in maternal and child health in urban India: a decompostition analysis. PLoS One.

[49] Ostlund U, Kidd L, Wengstrom Y, Rowa-Dewar N (2011). Combining qualitative and quantitative research with in mixed methods research designs: a methodological review. Int J Nurs Stud.

[50] Creswell JW, Plano CVL (2011). Designing and conducting mixed methods research.

[51] Guetterman TC, Fetters MD, Creswell JW (2015). Integrating quantitative and qualitative results in health science mixed methods research through joint displays. Ann Fam Med.

[52] Andrew S, Halcomb EJ (2006). Mixed methods research is an effective method of enquiry for community health research. Contemp Nurse.

[53] Jefferey MB (2004). Mixed methods studies: a foundation for primary care research. Ann Fam Med.

[54] Global Economic Prospects. The World Bank in India. Available at http://www.worldbank.org/en/publication/global-economic-prospects. Accessed 7 July 2017.

[55] National Health Policy 2017. Ministry of health and family welfare. Government of India. Available at http://cdsco.nic.in/writereaddata/National-Health-Policy.pdf. Accessed 7 Sept 2017.

[56] Windsor LC (2013). Using concept mapping in community-based participatory research: a mixed methods approach. J Mix Methods Res.

