# Supplementary material for: A mixed methods study on evaluating the performance of a multi-strategy national health program to reduce maternal and child health disparities in Haryana, India
Source: BMC Public Health. 2017 Sep 11;17:698. doi: 10.1186/s12889-017-4706-9 (PMC5594476; doi:10.1186/s12889-017-4706-9)

Additional Figure 1. The conceptual Framework of NRHM.

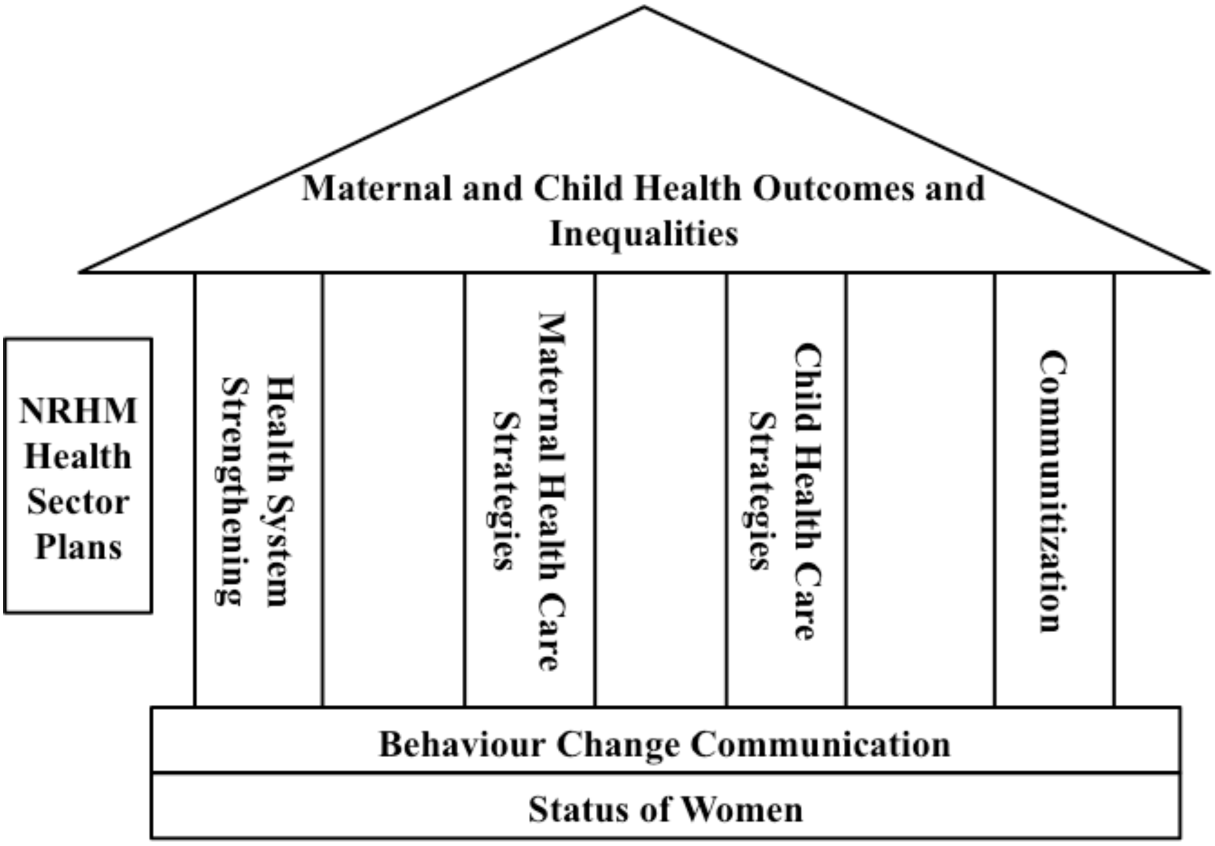

Additional Figure 2. Comparison of budget utilization rate of health system strengthening, communitization, maternal and child health care strategies components of National Rural Health Mission from 2007-08 to 2012-13.

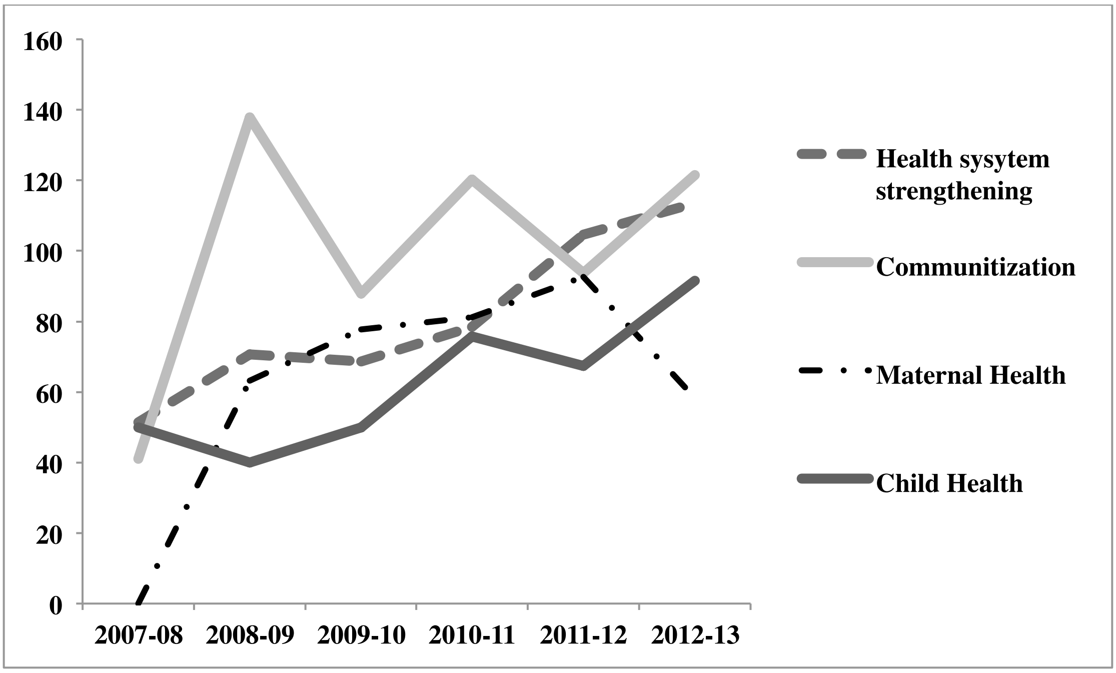

Additional Figure 3. Trend of budget utilization rate of strategies under health system strengthening component of National Rural Health Mission from 2007-08 to 2012-13.

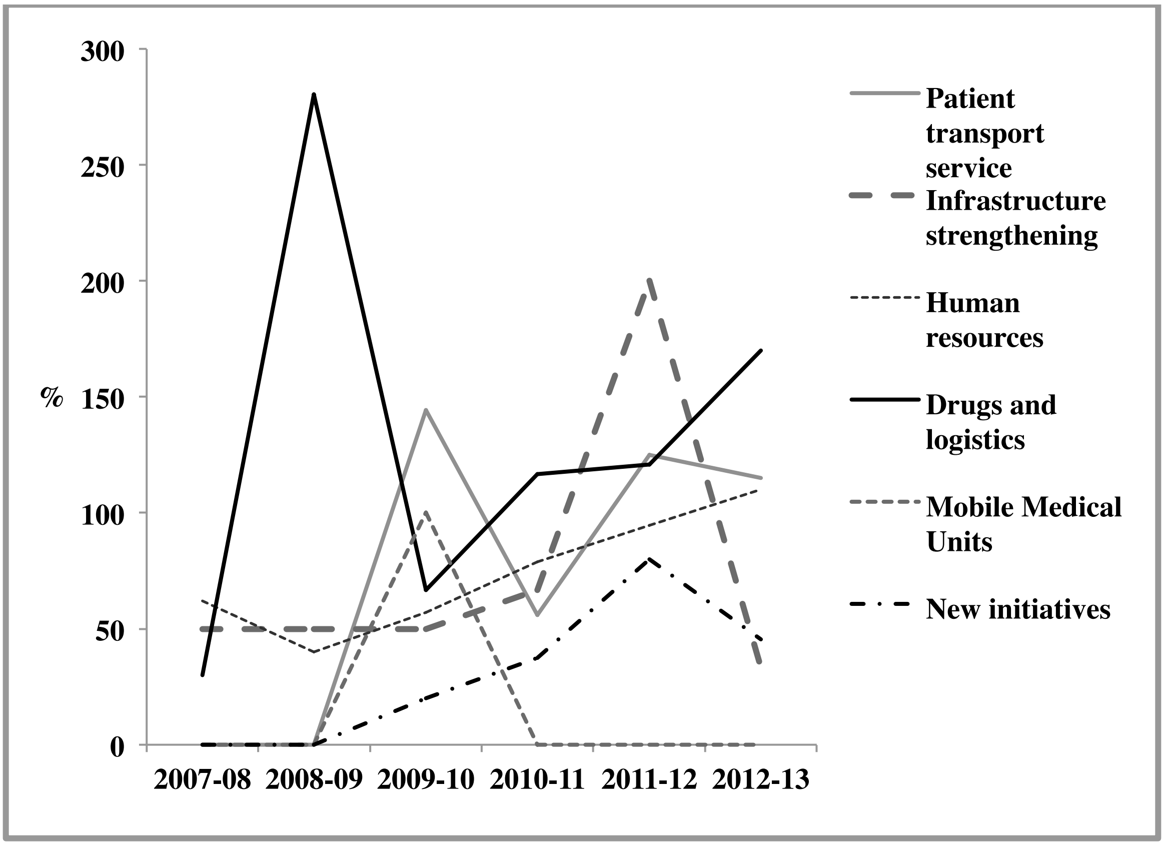

Additional Figure 4. Trend of budget utilization rate of strategies under communitization component of National Rural Health Mission from 2007-08 to 2012-13.

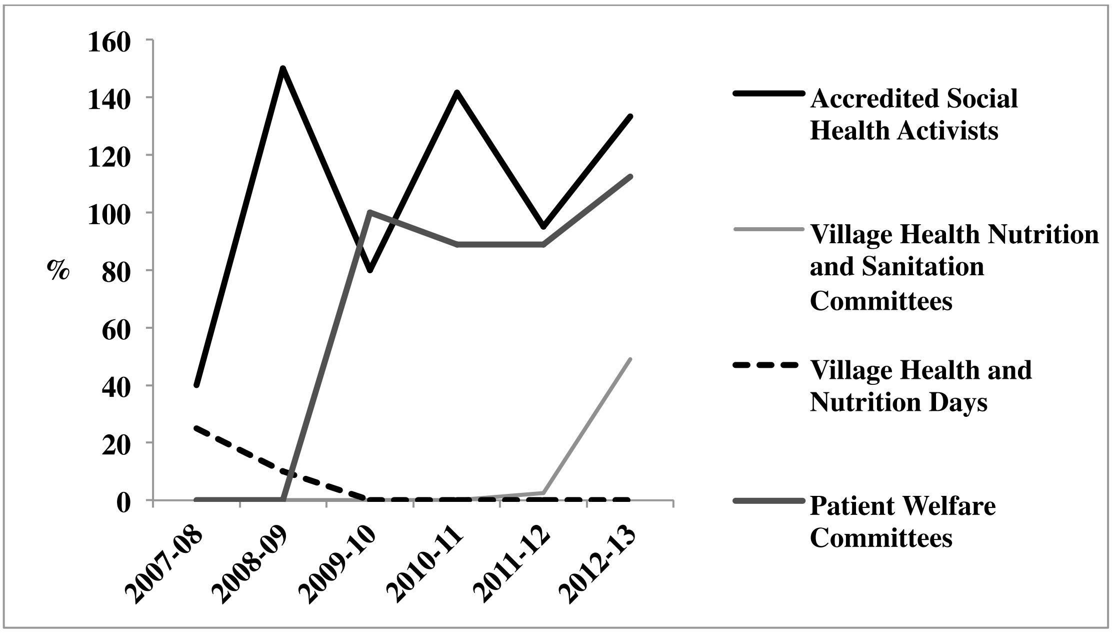

Additional Figure 5. Trend of budget utilization rate of maternal health care strategies of National Rural Health Mission from 2007-08 to 2012-13.

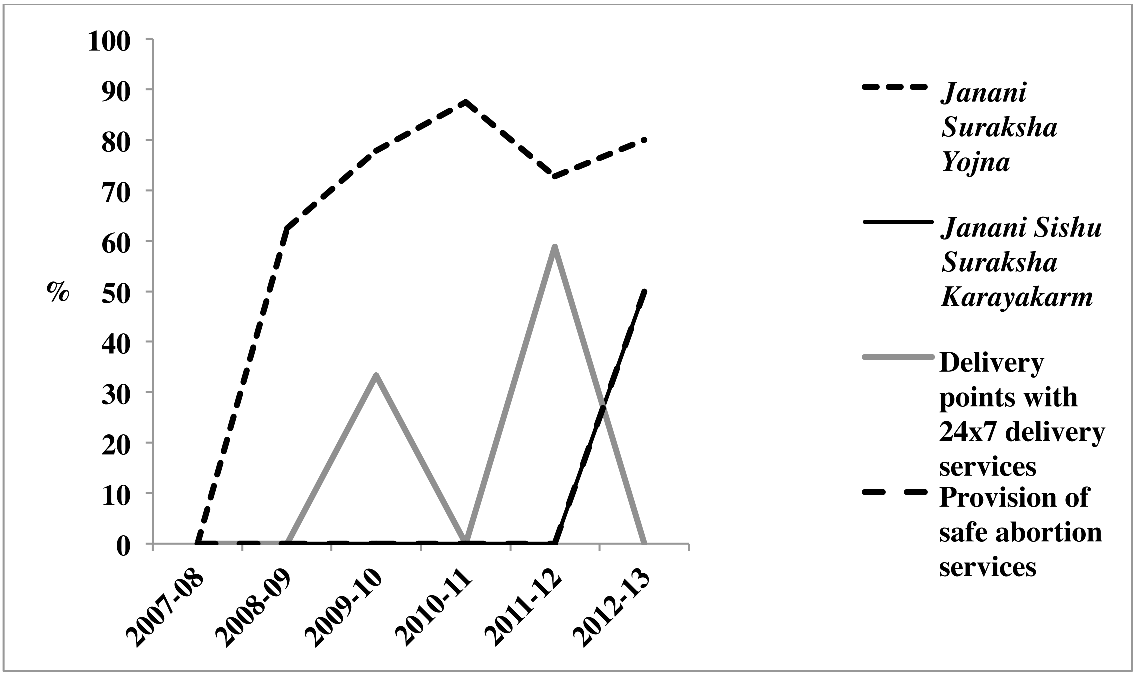

Additional Figure 6. Trend of budget utilization rate of child health care strategies component of NRHM from 2007-08 to 2012-13.

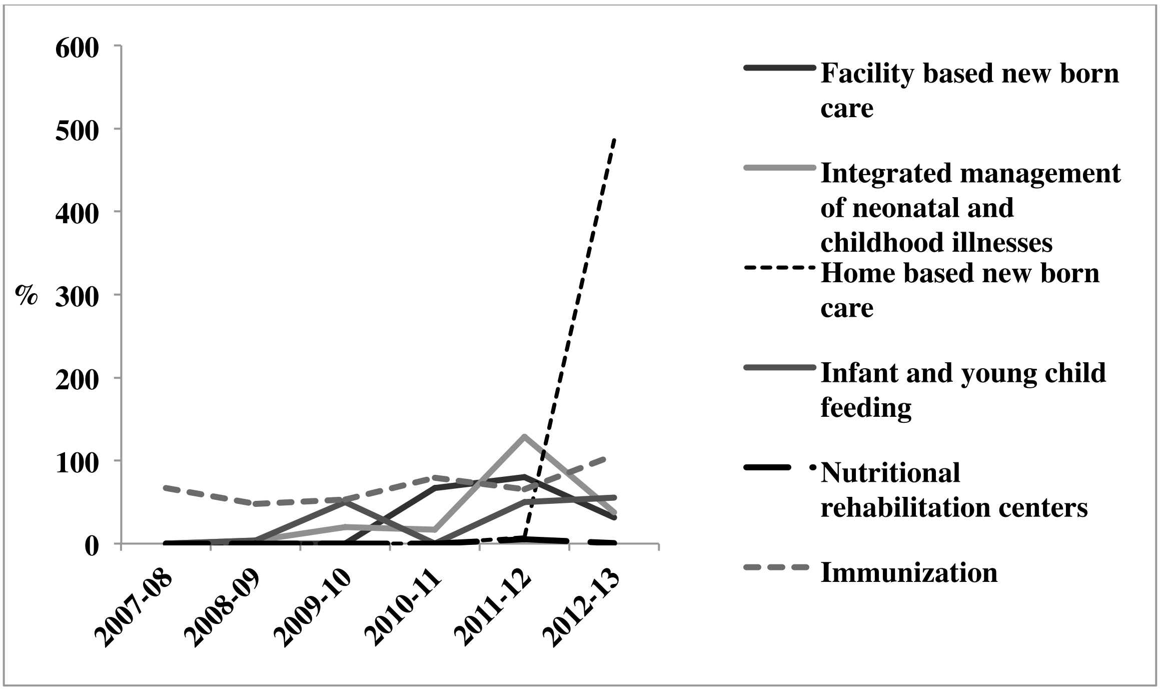

Additional Figure 7. The pathway of change as derived from theory of change.

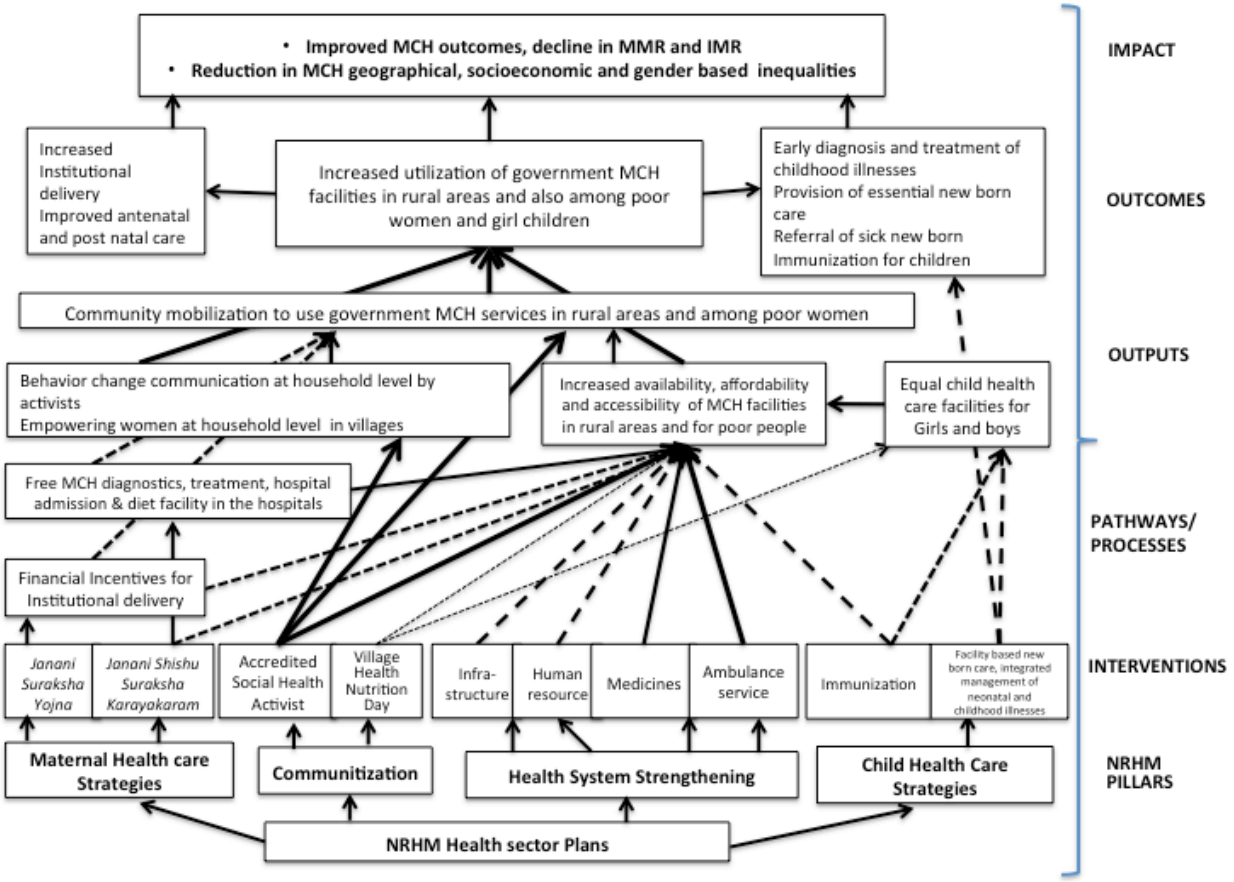

Supplement: Supplementary file 1 — The conceptual Framework of NRHM. Figure S2. Comparison of budget utilization rate of health system strengthening, communitization, maternal and child health care strategies components of National Rural Health Mission from 2007 to 08 to 2012–13. Figure S3. Trend of budget utilization rate of strategies under health system strengthening component of National Rural Health Mission from 2007 to 08 to 2012–13. Figure S4. Trend of budget utilization rate of strategies under communitization component of National Rural Health Mission from 2007 to 08 to 2012–13. Figure S5. Trend of budget utilization rate of maternal health care strategies of National Rural Health Mission from 2007 to 08 to 2012–13. Figure S6. Trend of budget utilization rate of child health care strategies component of NRHM from 2007 to 08 to 2012–13. Figure S7. The pathway of change as derived from theory of change. (PDF 300 kb) [file 12889_2017_4706_MOESM1_ESM.pdf]
