# Supplementary material for: A mixed methods study on evaluating the performance of a multi-strategy national health program to reduce maternal and child health disparities in Haryana, India
Source: BMC Public Health. 2017 Sep 11;17:698. doi: 10.1186/s12889-017-4706-9 (PMC5594476; doi:10.1186/s12889-017-4706-9)
Supplement: Supplementary file 2 — Year wise distribution of budget sanctioned, expenditure incurred (in million USD) and percentage of budget left unspent for NRHM’s maternal and child health sector plans from the financial year 2007–08 to 2012–13. (PDF 113 kb) [file 12889_2017_4706_MOESM2_ESM.pdf]

Additional Table 1. Year wise distribution of budget sanctioned, expenditure incurred (in million USD) and budget utilization rate (%) for NRHM's maternal and child health sector plans from the financial year 2007-08 to 2012-13.

NRHM Plans	2007-08			2008-09			2009-10			2010-11			2011-12			2012-13		
	S[Φ]	E[α]	%[β]	S	E	%	S	E	%	S	E	%	S	E	%	S	E	%
**1. Health system strengthening**	3.50	1.80	51.4	3.54	2.50	70.6	5.90	4.05	68.6	9.3	7.3	78.5	12.9	13.5	104.6*	13.3	15.1	113.5*
Patient transport service	0	0	0	0	0	0	0.9	1.3	144.4*	2.5	1.4	56	2.4	3	125	2	2.3	115*
Infrastructure development and strengthening	0.4	0.2	50	1	0.5	50	0.6	0.3	50	0.3	0.2	66.7	0.1	0.2	200*	0.3	0.1	33.3
Human resources	2.1	1.3	62	2	0.8	40	3.5	2	57.1	3.3	2.6	78.8	7.5	7.1	94.7	7.9	8.7	110*
Drugs and logistics	1	0.3	30	0.447	1.254	280.5*	0.3	0.2	66.7	2.4	2.8	116.7*	2.4	2.9	120.8*	2	3.4	170*
Mobile medical	0	0	0	0	0	0	0.1	0.1	100	0.1	0	0	0.1	0	0	0.1	0	0

units																		
New initiatives	0	0	0	0	0	0	0.5	0.1	20	0.8	0.3	37.5	0.5	0.4	80	1.1	0.5	45.5
**2. Communitization**	0.56	0.23	41.1	0.58	0.80	137.93*	1.90	1.67	87.89	2.1	2.5	120.2*	1.90	1.78	93.7	3.07	3.73	121.5*
Accredited Social Health Activists	0.5	0.2	40	0.6	0.9	150	1	0.8	80	1.2	1.7	141.7*	1	0.95	95	2.1	2.8	133.3*
Village health Nutrition and Sanitation Scheme	0	0	0	0	0	0	0	0	0	0	0	0	0.04	0.001	2.5	0.1	0.049	49
Village health and nutrition day	0.04	0.01	25	0.02	0.002	10	0	0.001	0	0	0	0	0	0	0	0	0	0
Patient welfare committees	0	0	0	0	0	0	0.9	0.9	100	0.9	0.8	88.9	0.9	0.8	88.9	0.8	0.9	112.5*
**3. Maternal Health Care Strategies**	0.0034	0	0	0.79	0.5	63.29	0.90	0.70	77.78	0.9	0.7	81.2	1.09	1.01	92.66	4.80	2.80	58.33

*Janani Suraksha Yojna^*	0.003	0	0	0.8	0.5	62.50	0.9	0.7	77.8	0.8	0.7	87.5	1.1	0.8	72.7	1	0.8	80
Janani Shishu Suraksha Karayakaram#	0	0	0	0	0	0.00	0	0	0	0	0	0	0	0.14	0	3.8	1.9	50
Delivery points with 24x7 delivery services	0	0	0	0.005	0	0.00	0.03	0.01	33.33	0	0	0	0.03	0.02	58.8	0	0.002	0
Provision of safe abortion services	0	0	0	0	0	0.00	0	0	0.00	0.04	0	0	0	0.001	0	0.004	0.002	50
**4. Child Health Care Strategies**	0.36	0.18	50	0.55	0.22	40	0.80	0.40	50	2.8	2.1	75.72	1.44	0.97	67.36	3.87	3.54	91.47
Facility based new born care	0	0	0	0	0	0.00	0	0	0.00	0.3	0.2	66.7	0.5	0.4	80	0.48	0.15	31.3

Integrated management of neonatal and childhood illnesses	0.03	0	0	0.03	0.001	3.33	0.05	0.01	20.00	0.03	0.005	16.7	0.03	0.04	129	0.032	0.012	37.5
Home based new born care	0	0	0	0	0	0.00	0	0	0.00	0	0	0	0.13	0.01	7.7	0.007	0.034	486*
Infant and young child feeding	0.001	0	0	0.05	0.002	4.00	0.04	0.02	50.00	0	0	0	0.02	0.01	50	0.009	0.005	55.6
Nutritional rehabilitation centers	0	0	0	0	0	0.00	0.003	0	0.00	0	0	0	0.004	0.0002	5	0.017	0.0001	0.6
Immunization	0.3	0.2	66.67	0.46	0.22	47.83	0.7	0.37	52.86	2.4	1.9	79.2	0.75	0.49	65.3	3.1	3.3	106*

α sanctioned; β Expenditure done; γ Budget Utilization Rate; *Extra budget is received from state budget; ^*Janani Suraksha Yojna*- it was a financial incentive scheme for pregnant women to increase the institutional delivery rate:# *Janani Shishu Suraksha Karayakaram*- This scheme was launched to provide cashless delivery services for pregnant women including antenatal, natal and postnatal diagnostic, curative and transport services, and free treatment of sick infants in the public health facilities.
