# Supplementary material for: A mixed methods study on evaluating the performance of a multi-strategy national health program to reduce maternal and child health disparities in Haryana, India
Source: BMC Public Health. 2017 Sep 11;17:698. doi: 10.1186/s12889-017-4706-9 (PMC5594476; doi:10.1186/s12889-017-4706-9)
Supplement: Supplementary file 3 — Extent of Implementation on NRHM’s maternal and child health sector plans in Haryana. (PDF 69 kb) [file 12889_2017_4706_MOESM3_ESM.pdf]

Additional Table 2. Status of Implementation on NRHM's maternal and child health sector plans in Haryana.

NRHM Plans	Extent of Implementaion (Budget Utilization Rate)				
	Full	Partial			Nil
		High-level	Mid-level	Low-level	
	(>=100%)	(80-99%)	(20-79%)	(1-19%)	(<1%)
**1. Health System Strengthening**	✔	-	-	-	-
Patient transport service/referral services	✔	-	-	-	-
Infrastructure development and strengthening	-	-	✔	-	-
Human resources	✔	-	-	-	-
Drugs and logistics	✔	-	-	-	-
Mobile medical units	-	-	-	-	✔
New Initiative	-	-	✔	-	-
**2. Communitization**	✔	-	-	-	-
Accredited Female Health Activist	✔	-	-	-	-
Village health and sanitation committees	-	-	✔	-	-
Village health and nutrition days	-	-	-	-	✔
Patient welfare committees	✔	-	-	-	-
**3. Maternal Health Care Strategies**	-	-	✔	-	-
Janani Suraksha Yojna	-	✔	-	-	-
Janani Shishu Suraksha Karyakaram	-	-	✔	-	-
Delivery points with provision of 24x7 delivery services	-	-	-	-	✔
Provision of safe MTP services	-	-	✔	-	-
**4. Child Health Care Strategies**	-	✔	-	-	-

Facility based new born care	-	-	✔	-	-
Integrated management of childhood illnesses	-	-	✔	-	-
Home based new born care	✔	-	-	-	-
Infant and young child feeding	-	-	✔	-	-
Nutrition Rehabilitation Centers	-	-	-	-	✔
Immunization	✔	-	-	-	-
