# Supplementary material for: A mixed methods study on evaluating the performance of a multi-strategy national health program to reduce maternal and child health disparities in Haryana, India
Source: BMC Public Health. 2017 Sep 11;17:698. doi: 10.1186/s12889-017-4706-9 (PMC5594476; doi:10.1186/s12889-017-4706-9)
Supplement: Supplementary file 4 — Status of maternal and child health indicators pre, during and post NRHM implementation in Haryana as per DLHS rounds 2, 3 and 4. (PDF 106 kb) [file 12889_2017_4706_MOESM4_ESM.pdf]

Additional Table 3. Status of maternal and child health indicators pre, during and post NRHM implementation in Haryana as per DLHS rounds 2, 3 and 4.

**Indicators**	**Pre NRHM**	**During NRHM**	**Post NRHM**	**P value**
	**(2002-2004)**	**(2007-2008)**	**(2012-2013)**	
Maternal Mortality Ratio	1.86	1.53	1.21	0.13
Infant Mortality Rate	59	55	40	0.09
**Antenatal care (%): Pregnant women**				
Registered in the first trimester	13.7	55.1	82.1	0.00
With three or more ANC	43.1	51.9	74.5	0.00
With full ANC check up	11.8	13.3	21.8	0.06
Who got at least one TT injection	83.5	86.1	93.6	0.04
Two TT injections	77.5	79.4	58.1	0.05
Who had atleast 100 IFA tablets	16.5	29.0	29.5	0.00
**Natal care** (%)				
Institutional delivery rate	35.7	46.9	76.9	0.00
Safe deliveries (Deliveries assisted by skilled birth attendants)	43.9	53.4	91.0	0.00
**Post natal care (%): Mothers who received post natal care within**				
48 hours of delivery	NA	48.8	67.2	0.01
2 weeks of delivery	8.9	49.5	69	0.07
**Child Health (%):Children aged 12-23 months who received**				
Full immunization	59.1	59.6	52.1	0.28
No vaccination	11.8	1.9	5.9	0.00
BCG vaccine	83.5	86.5	84.2	0.96

3 doses of DPT vaccine	72.9	67.9	71.1	0.72
3 doses of polio vaccine	73.6	69.0	72.7	0.83
Measles vaccine	65.4	69.0	70.0	0.53
**Breast feeding practices (%)**				
Exclusively breast fed for atleast 6 months	33	5.7	27.4	0.00
**Women awareness about**				
Diarrhoea Managment	49.8	79	81.7	0.00
Danger signs of Acute respiratory infection	49.8	76.3	75.2	0.00
**Percentage of women whose child suferred from**[¥]				
Diarrhoea	18.1	16.0	4.0	0.00
ARI	10.8	8.3	3.6	0.00
**Childhood Diseases: Children with (illness reported in last two weeks)**				
Diarrhoea who received ORS	32.3	31.7	44.8	0.08
Diarrhoea who sought advise/treatment	78.4	82.0	68.7	0.05
Acute respiratory infection who sought advise/treatment	78	88.1	85.5	0.5
