# Supplementary material for: A mixed methods study on evaluating the performance of a multi-strategy national health program to reduce maternal and child health disparities in Haryana, India
Source: BMC Public Health. 2017 Sep 11;17:698. doi: 10.1186/s12889-017-4706-9 (PMC5594476; doi:10.1186/s12889-017-4706-9)
Supplement: Supplementary file 5 — Geographical, socioeconomic and gender inequalities in maternal and child health indicators pre, during and post NRHM implementation (expressed as absolute difference in proportion of indicators). (PDF 106 kb) [file 12889_2017_4706_MOESM5_ESM.pdf]

Additional Table 4. Geographical, socioeconomic and gender inequalities in maternal and child health indicators pre, during and post NRHM implementation (expressed as absolute difference in proportion of indicators).

Geographical Inequalities	Pre NRHM (2002-2004)			During NRHM (2007-2008)			Post NRHM (2012-2013)			P value[#]
	Rural	Urban	Diff	Rural	Urban	Diff	Rural	Urban	Diff	
**Maternal Health: Women who had (%)**										
Three or more ANC	40.8	63.9	23*	47.2	66.1	18.9*	72.4	77.8	5.4*	0.00
Full ANC check up	9.5	17.9	8*	10.2	22.6	12.4*	19.2	26.0	6.8*	0.00
Received two TT injections	75	84.3	9.3*	76.9	87	10.1*	54.4	65.2	10.8*	0.02
Consumed IFA for at least 3 months	15.9	21.6	6*	28.1	31.7	3.6*	27.6	32.6	5*	0.08
Institutional delivery rate	27.3	56.4	29*	42.2	61.4	19.2*	74.3	81.9	7.6*	0.00
PNC with in 2 weeks of delivery	9.6	6.8	2.8*	46.5	58.7	12.2*	68.5	70.0	1.5	0.00
**Child Health (%)**										
Children age 12-23 months who received Full vaccination	56.7	66.3	10*	55.9	70.8	14.9*	51.0	54.5	3.5	0.00

Children with diarrhoea who received ORS	29	40	11*	28.3	44.2	15.9*	45.6	43.4	-2.2	0.01
Infant Mortality Rate (per thousand live births)	66	47	19*	60	44	16*	44	32	12*	0.09
**Socioeconomic Inequalities**	**Standard of living index**			**Wealth Index**			**Wealth Index**			**P value**
	**Low**	**High**	**Diff**	**Lowest**	**Highest**	**Diff**	**Lowest**	**Highest**	**Diff**	
**Maternal Health: Women who had (%)**										
Three or more ANC	16.8	17	0.2	16.3	72.4	46.5*	57	80	23*	0.00
Full ANC check up	3.6	20.1	16.5*	1.4	23.5	20.1*	20	36	16*	0.00
Received two TT injections	58.5	88.5	30.3*	48.5	91.8	35.6*	77	84	7*	0.00
Consumed IFA for at least 3 months	8.5	25.4	16.9*	2.2	28.1	22.3*	30	44	14*	0.00
Institutional delivery rate	11.8	60	48.2*	14.8	70.9	52.5*	75	88	13*	0.00
PNC with in 2 weeks of delivery	6.4	8.6	2.2*	26.9	66.7	39.8*	55.4	72.1	16.7*	0.00
**Child Health (%)**										
Children (age 12-23 months) who received Full	30.6	78.9	48.3*	28.5	74.4	44.4*	62	76	14*	0.00

vaccination										
Children with diarrhoea who received ORS	7.8	48.5	40.7*	10.3	44.7	34.4*	32	37	5	0.00
**Gender Inequalities**	**Male**	**Female**	**Diff**	**Male**	**Female**	**Diff**	**Male**	**Female**	**Diff**	**P value**
	**n=1221**	**n=993**		**n=1178**	**n=947**		**n=1142**	**n=896**		
Full Immunization	61.8	56.1	5.7*	62.5	56.0	6.5*	51.9	52.5	-0.6	0.00
No Vaccination	11.3	12.7	-1.4	1.7	2.1	-0.4*	6.5	6.8	-0.3	0.00
BCG vaccine	84.3	82.4	1.9	87.8	84.9	2.9*	83.8	84.7	-0.9	0.00
3 doses DPT vaccine	75.6	70.7	4.9	71.7	65.8	5.9*	72.2	73.5	-1.3	0.00
3 doses of OPV vaccine	74.7	70.1	4.6*	70.2	64.7	4.6*	71.1	71.1	0	0.00
Measles vaccine	67.6	62.2	4.2*	70.9	66.7	5.4*	69.8	69.7	0.1	0.00

*p<0.05; diff=Difference; # indicates statistically significant difference in the inequality across time periods
