# Supplementary material for: A mixed methods study on evaluating the performance of a multi-strategy national health program to reduce maternal and child health disparities in Haryana, India
Source: BMC Public Health. 2017 Sep 11;17:698. doi: 10.1186/s12889-017-4706-9 (PMC5594476; doi:10.1186/s12889-017-4706-9)
Supplement: Supplementary file 6 — Themes and codes as per applied thematic analysis. (PDF 81 kb) [file 12889_2017_4706_MOESM6_ESM.pdf]

Additional Table 5. Themes and codes as per applied thematic analysis.

Themes	Code Family	Code names	
		**Predetermined codes**	**Emergent codes**
**Health system strengthening**	**Infrastructure**	Health centers in rural areas	Well equipped health centers in rural areas, non availability of waiting halls for patients in the health facilities, availability of clean health centers
	**Drugs and Logistics**	Medicines in health centers in rural areas	Free availability of medicines in health centers in rural areas; quality is an issue; stock out of situations
	**Patient Transport Service**	Ambulance service	Free availability of ambulance service; Issues with its maintenance, better services with in the ambulance needed at par with private, ambulance contact number could not be reached possibly due to frequent callers; late arrivals to the homes, inadequate number of vehicles; linked to increase in institutional delivery
	**Human resource**	Availability of doctors, nurses, midwives	Acute shortage of manpower especially specialist, contractual staff available but quality of contractual staff is an issue, salary of contractual staff not at par with regular staff, negative attitude of doctors, specialists not evenly distributed with in the state
	**Untied funds**	Availability of untied	Availability of funds for upgrading infrastructure as per the need, buying drugs as

		funds	per need, Availability of funds for arranging refreshments for mothers during mother meeting
	**Mobile Medical Units**	Availability of mobile medical units	Functional status of mobile medical units an issue, non availability of doctors, limited awareness of mobile medical units in the villages
**Communitization**	**Accredited Social Health Activists**	Availability of Accredited Social Health Activists in the villages	Role in immunization of children and pregnant women, improving institutional delivery, generating awareness about NRHM schemes & importance of institutional delivery Accompanies the families while travelling to the hospital; insufficient number; educational qualification has a bearing on recruitment of accredited social health activists; well known in the villages; good rapport with the women, especially decision makers (mother in laws); calls free ambulance; Community Mobilizer
	**Village Health and Nutrition Day**	Celebration of village health and nutrition days in the villages	Immunization sessions held on village health and nutrition days; mother meetings also held on these days; Known popularly as village health '*mela*'; Not held regularly

	**Village Health Nutrition & Sanitation Committee**	Formation of Village Health Nutrition & Sanitation Committee	Less awareness by mothers and community members, members are not involved in planning; village head would ask for bribe for utilizing the funds, funds remain unutilized; anganwadi worker involvement in funds handling leading to underutilization
**Maternal Health Care Strategy**	***Janani Suraksha Yojna***	Financial incentive for institutional delivery	Funds remain unutilized; Delay in payment due to administrative reasons; Lack of knowledge imparted to the mothers about the scheme; Linked with opening of bank accounts leading to issue in delivering the benefits to women who do not have bank accounts; Proofs required to get the benefits; Linked with increase in institutional delivery
	Janani Shishu Suraksha Yojna	Free medicine and institutional delivery	Free diet during hospital stay; Implementation is partial due to lack of adequate manpower; Linked with increased institutional delivery
**Child health care strategies**	**Immunization**	All children getting vaccines	Lack of sufficient auxiliary nurse midwives leads to partial implementation of immunization sessions; Cultural barrier are there for immunization of children especially in district Mewat; Fear of injections; accredited social health activists an catalyst in providing immunization in the form of mobilizing the community

	**Facility based newborn care**	Newborn care services in Government facilities	New born referred for treatment to government hospitals from private health facilities as government new born facilities are better
	**Integrated management of neonatal and childhood illnesses**	Treatment of sick children as per Integrated management of neonatal and childhood illnesses	Staff is trained in Integrated management of neonatal and childhood illnesses implementation; Community lack trust on government facilities for treatment of sick children so do not visit subcenters in villages for treatment (less demand at subcenter level); Lack of supervision; Poor implementation; Focus has been shifted from Integrated management of neonatal and childhood illnesses to home based post natal care
**MCH Inequalities**	**Geographical Inequality**	MCH inequalities in urban and rural areas	Increase in antenatal registrations in rural areas, gap is bridged with more villagers utilizing services than urban people due to NRHM. Awareness has improved and medicines are available in villages however facilities are still more in cities.
	**Socioeconomic Inequality**	MCH inequalities between rich and poor	Socioeconomic inequalities have decreased to some extent because of availability of free ambulances, medicines, diet during hospital stay for the poor. Food security in general would reduce this.
	**Gender Inequality**	Child health inequalities between girls and boys	NRHM has no scheme for targeting gender inequality; Small size of the families and increased educational status has led to the changes in gender inequality; Gender

			inequality is less seen in Mewat district
**Barriers**	**Client level**	-	Poor awareness about schemes provided under NRHM; Unmet basic need (lack of food) of pregnant women and mothers in rural areas; Lack of faith in government health facilities; Overriding household responsibilities of mothers; Poor health seeking behavior: Phobic towards pills/medicines/operation
	**Community Level**	-	Mother-in-law and male spouse are the potential influencers; Gender disparity in providing child care; Negative image among families about the quality of free services; Community norm and cultural belief; Lack of family planning discourages ensuring child health; Public attitude: Lack of willingness to wait; Low social status of women in the society
